# Supplementary material for: Dietary Impacts on Changes in Diversity and Abundance of the Murine Microbiome during Progression and Treatment of Cancer
Source: Nutrients. 2023 Jan 31;15(3):724. doi: 10.3390/nu15030724 (PMC9920799; doi:10.3390/nu15030724)
Supplement: Supplementary file 1 [file nutrients-15-00724-s001.zip › nutrients-1945700-supplementary.pdf]

Supplementary Materials

(a)

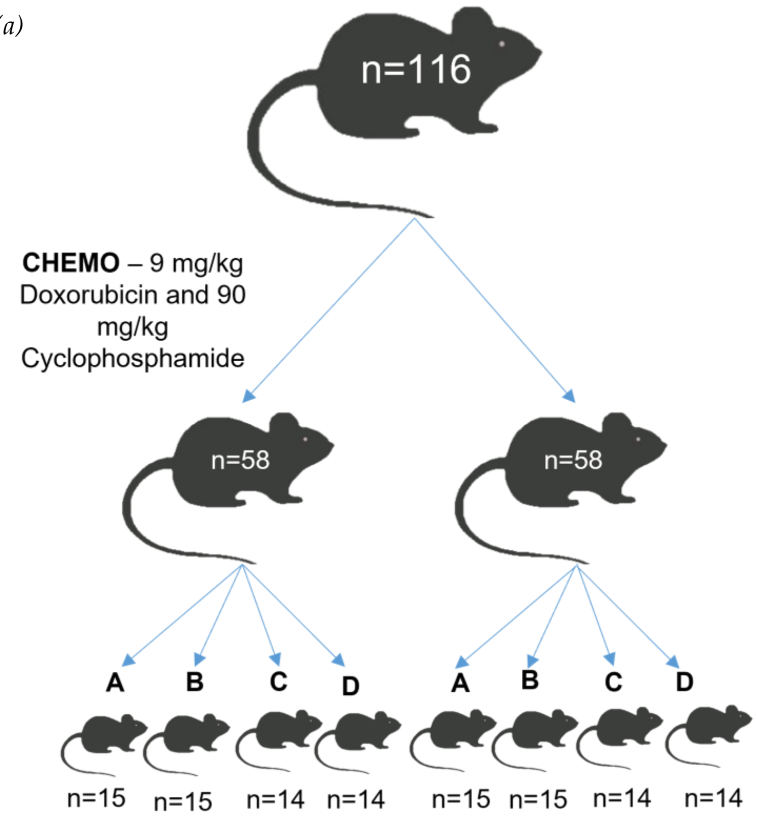

(b)

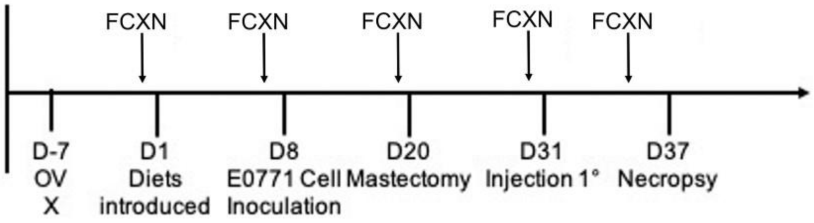

(c)

DIETS:

A low sucrose, high omega3 (2%kcal; EPA/DHA 1.5:1) – D15082608

B low sucrose, low omega3 (Low ALA-0% EPA/DHA) – D17011201

C high sucrose, high omega3 (2% kcal; EPA/DHA 1.5:1) – D15082609

D high sucrose, low omega3 (Low ALA-0% EPA/DHA) – D17091801

Figure S1. Experimental design for (a) assigning mice to cohorts, (b) experiment timeline and fecal collection points, and (c) dietary macronutrient balance

(a)

(b)

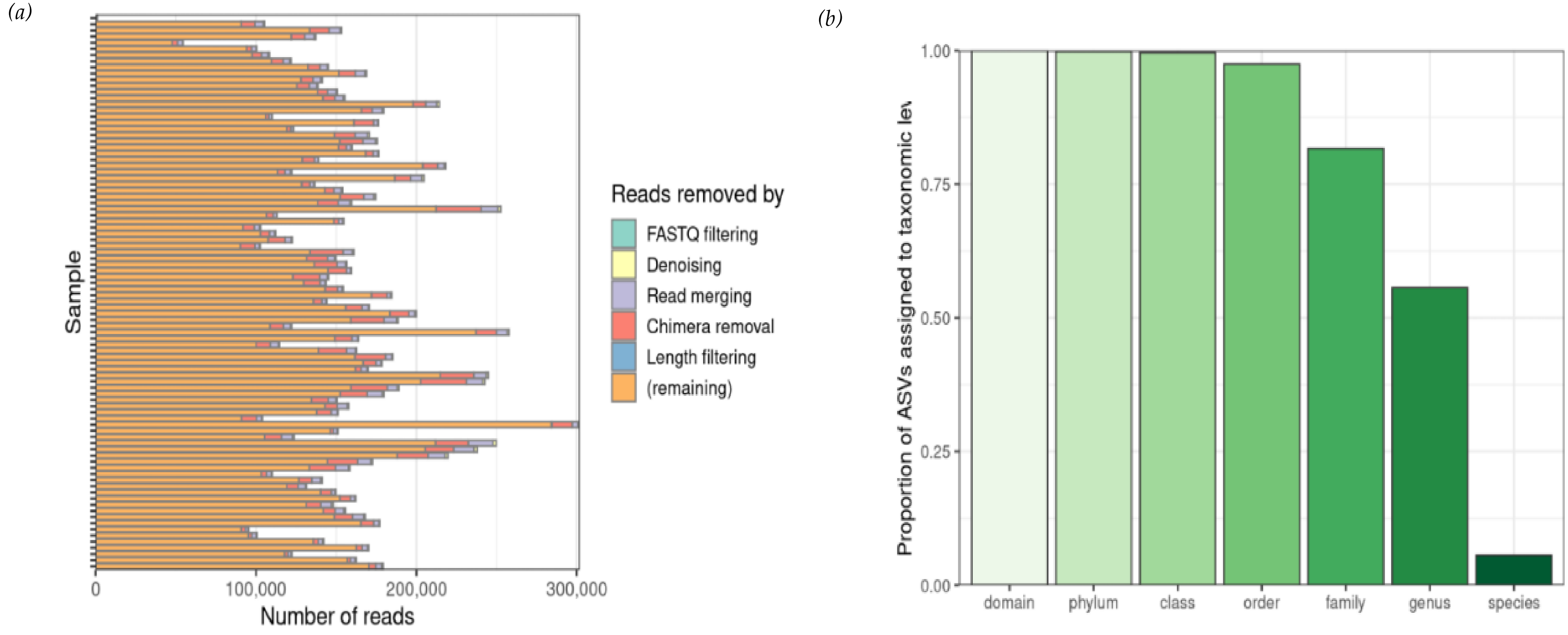

Figure S2. Quality control and sample processing (a) filtering and read removal along the data processing pipeline (b) proportion of ASVs assignments by taxonomic level

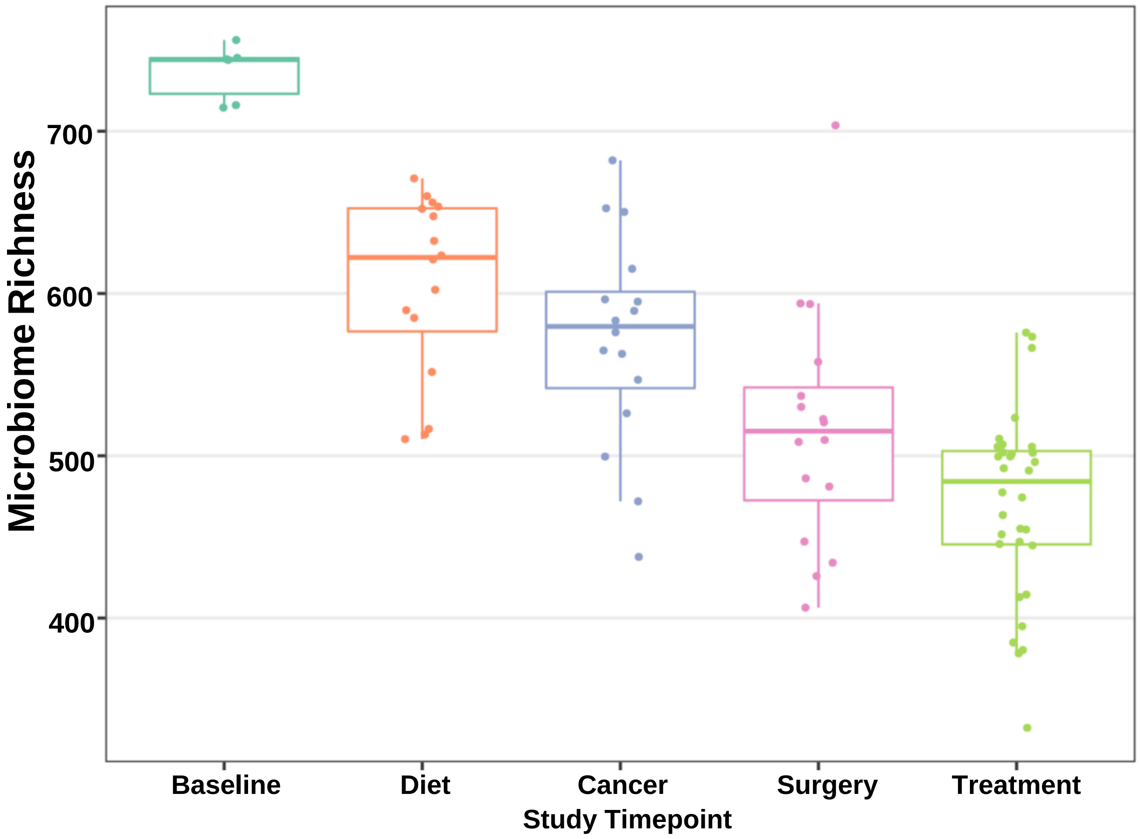

Figure S3a. Estimated richness over progression of disease and treatment. Microbiome population richness significantly decreased over time

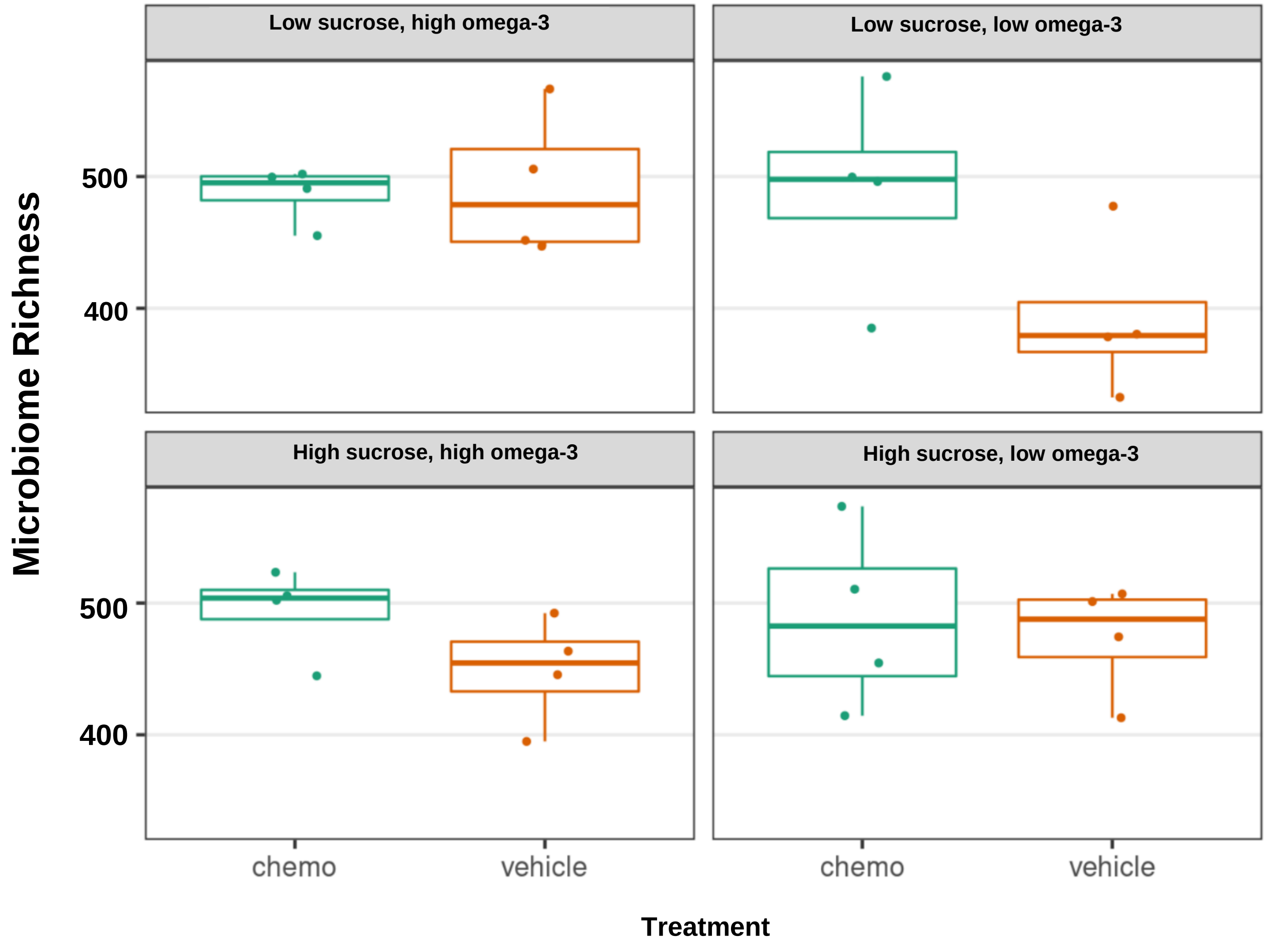

Figure S3b. Estimated richness by diet and treatment. Microbiome population richness differed by diet and treatment interactions

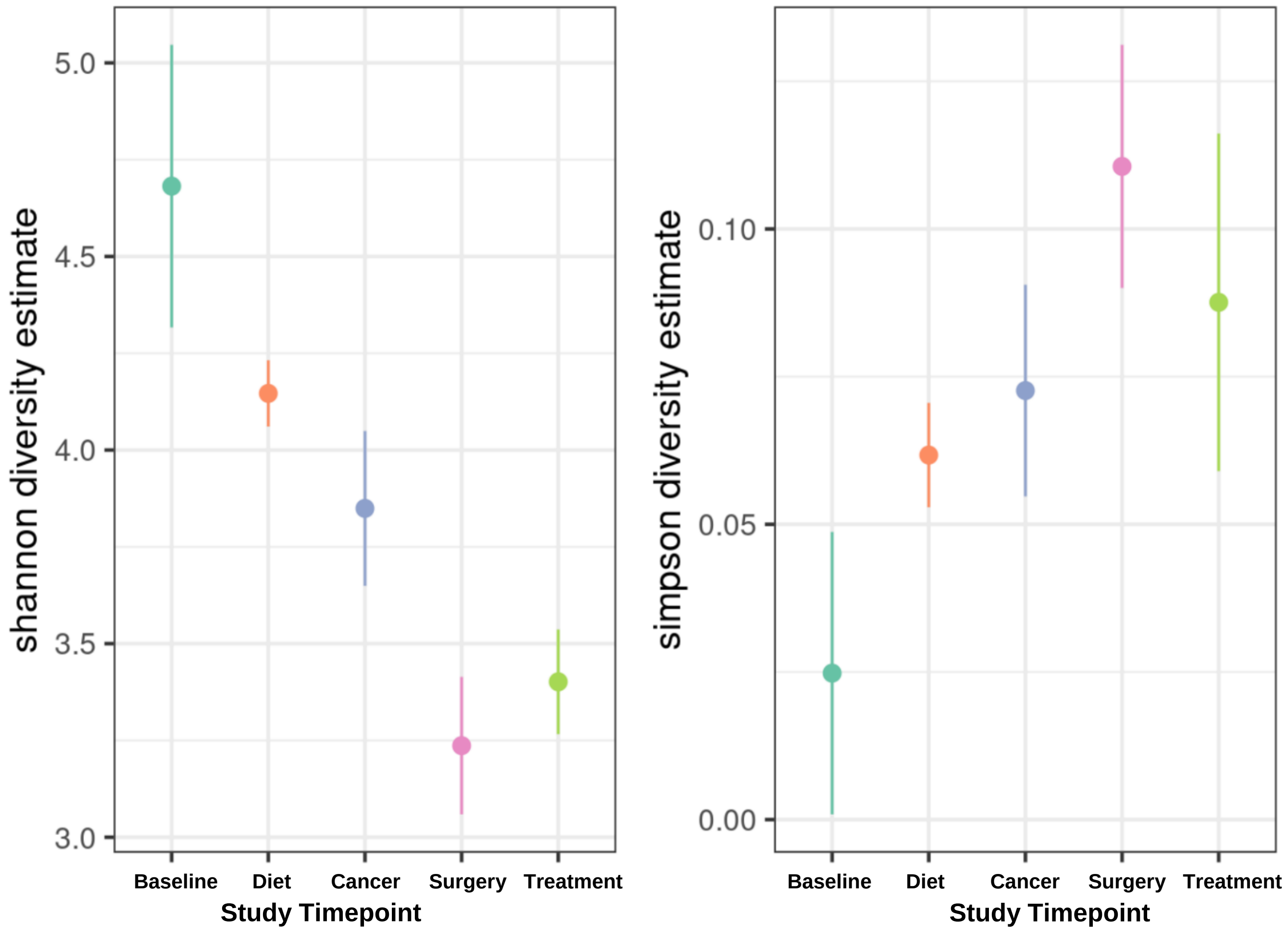

Figure S4a. Richness and evenness of ASV/per-sample, weighted by the Shannon and Simpson diversity indices, demonstrate differences in alpha-diversity modeled by study timepoint

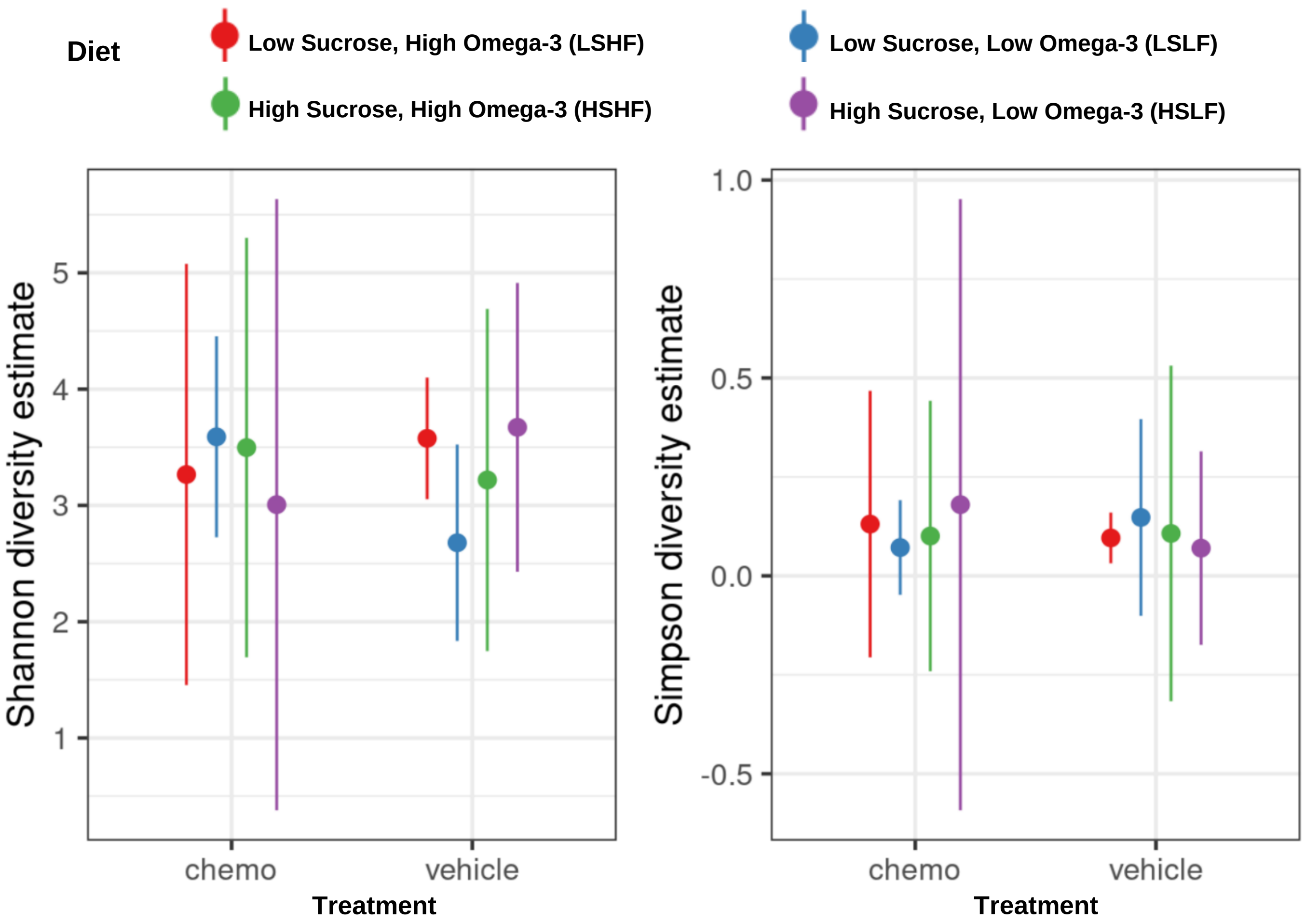

Figure S4b. Richness and evenness of ASV/per-sample, weighted by the Shannon and Simpson diversity indices, demonstrate differences in alpha-diversity modeled by diet and treatment

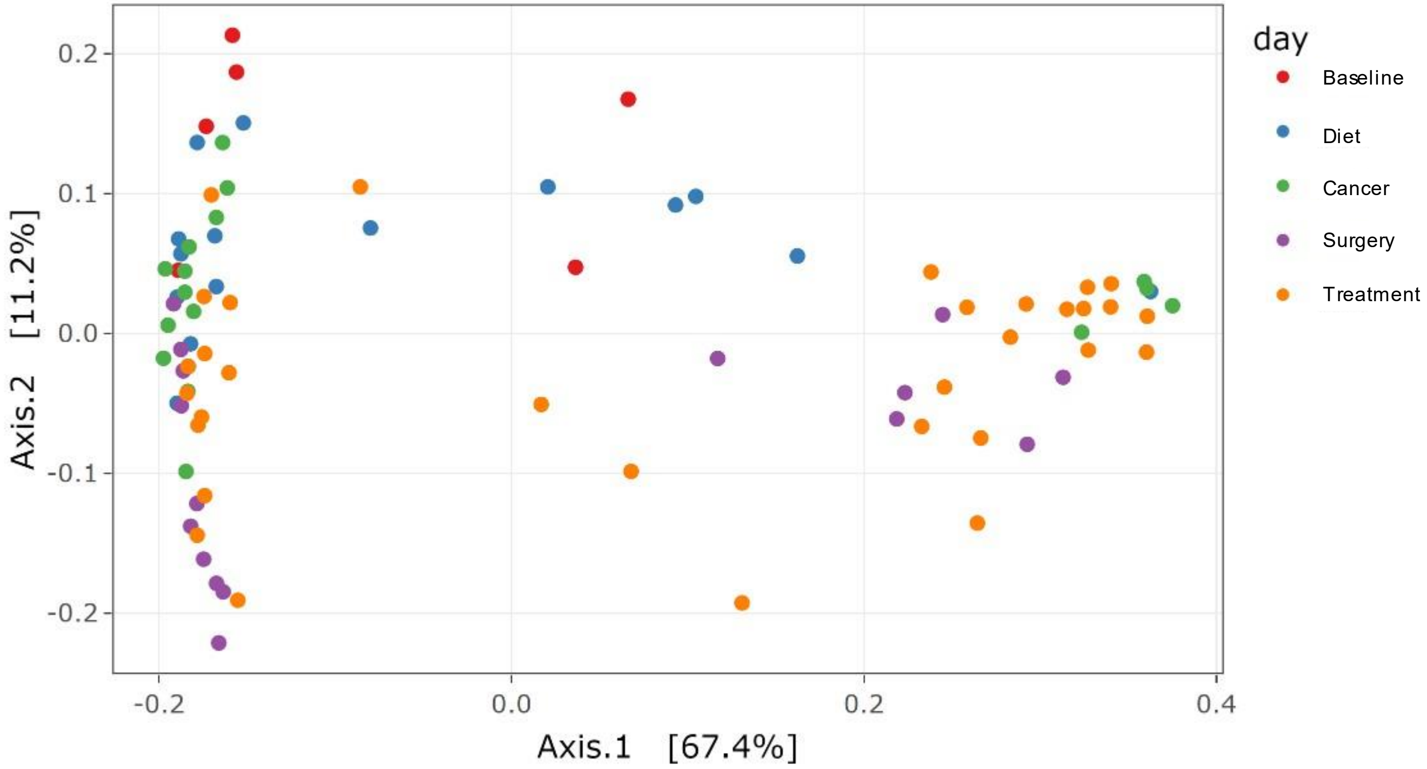

Figure S5a. Ordination from principal coordinate analysis which includes ASV1

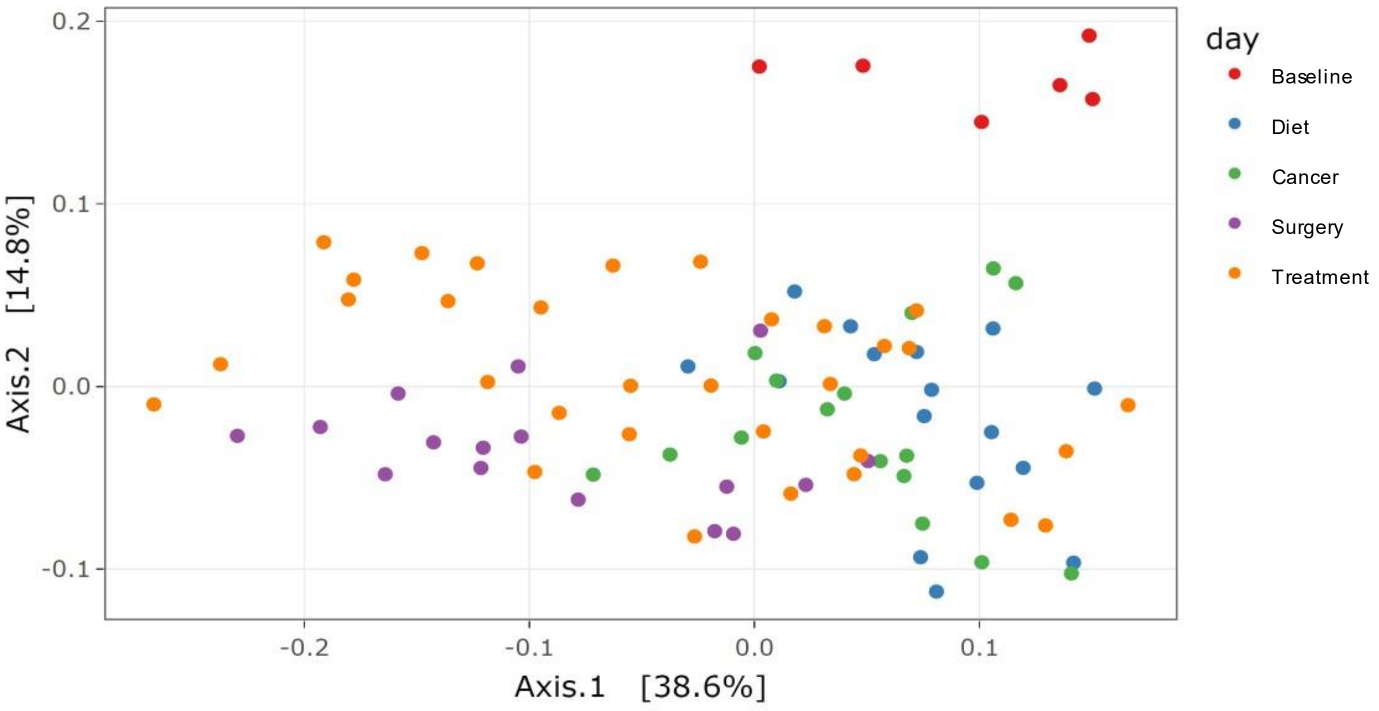

Figure S5b. Ordination from principal coordinate analysis which excludes ASV1

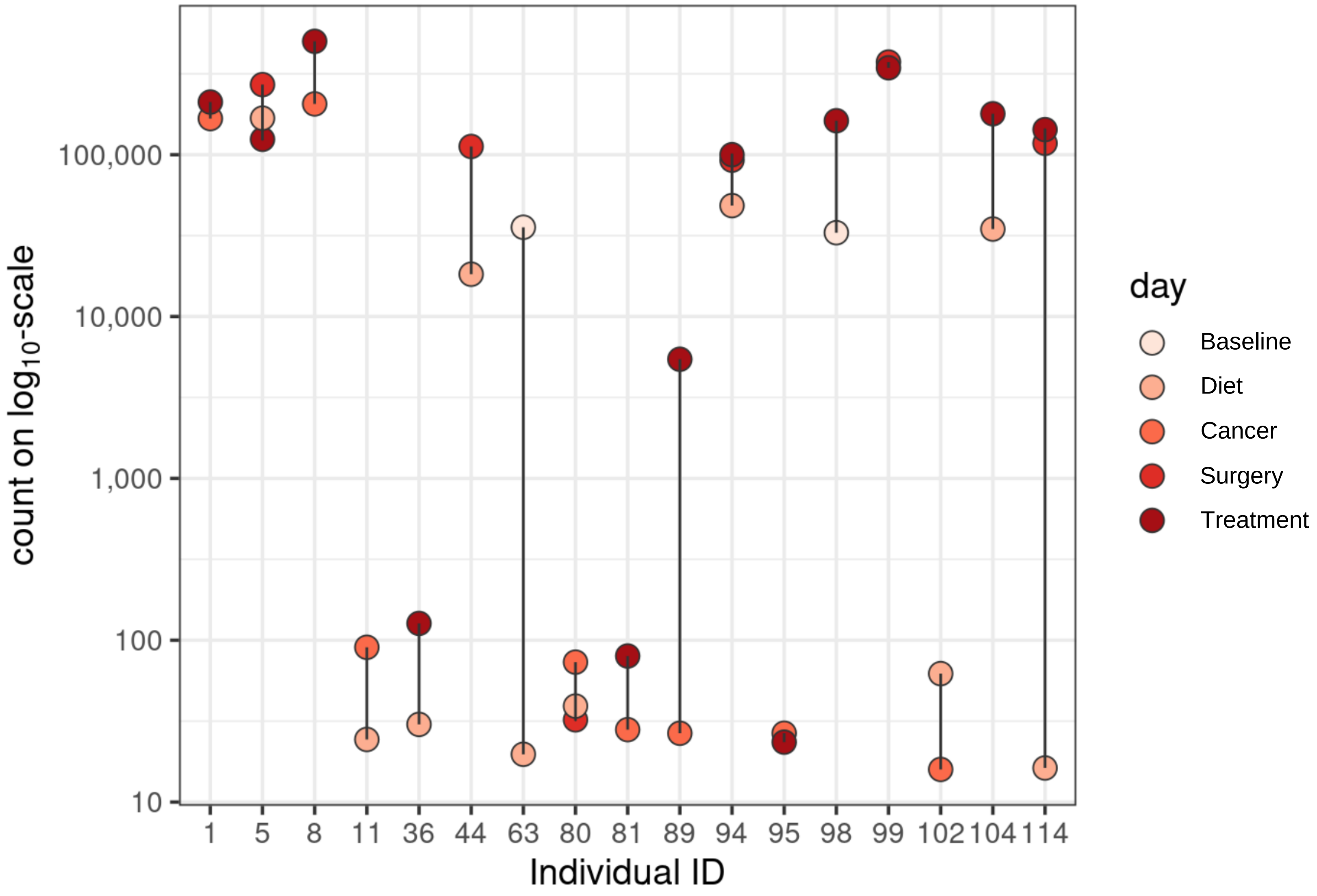

Figure S6. ASV1 abundance of individual mice by timepoint
